# Relationship between Aspiration Pneumonia and Feeding Care among Home Care Patients with an In-Dwelling Nasogastric Tube in Taiwan: A Preliminary Study

**DOI:** 10.3390/ijerph19095419

**Published:** 2022-04-29

**Authors:** Szu-Yu Hsiao, Ching-Teng Yao, Yi-Ting Lin, Shun-Te Huang, Chi-Chen Chiou, Ching-Yu Huang, Shan-Shan Huang, Cheng-Wei Yen, Hsiu-Yueh Liu

**Affiliations:** 1 School of Dentistry, College of Dental Medicine, Kaohsiung Medical University, Kaohsiung 807378, Taiwan; szyuhs@kmu.edu.tw (S.-Y.H.); 106820001@kmu.edu.tw (Y.-T.L.); 2 Division of Pediatric and Special Needs Dentistry, Department of Dentistry, Kaohsiung Medical University Hospital, Kaohsiung 807377, Taiwan; shunteh@kmu.edu.tw (S.-T.H.); 1010396@kmuh.org.tw (C.-Y.H.); 1070480@kmuh.org.tw (S.-S.H.); 1040474@kmuh.org.tw (C.-W.Y.); 3 Master Program of Long-Term Care in Aging, Kaohsiung Medical University, Kaohsiung 80708, Taiwan; angusyao@kmu.edu.tw; 4 Department of Oral Hygiene, College of Dental Medicine, Kaohsiung Medical University, Kaohsiung 807378, Taiwan; a0983878357@gmail.com; 5 Chi-Mei Medical Center, Tainan 710402, Taiwan; 6 Department of Medical Research, Kaohsiung Medical University Hospital, Kaohsiung Medical University, Kaohsiung 807377, Taiwan

**Keywords:** long-term care, oral function, swallow, elderly, caregiver

## Abstract

Home care patients have swallowing dysfunction and rely on an in-dwelling nasogastric tube (NGT) to complement oral food intake, supplement their diet, and maintain adequate nutritional status. This study explored the relationship between aspiration pneumonia (AP) and feeding care among home care patients with an in-dwelling NGT. This preliminary study employed a cross-sectional design. There were 35 patients who relied on an in-dwelling NGT to complement their oral intake of food (NGT-oral feeding) and their primary caregivers participated in this study. All of them developed AP in the past year. Factors involving food intake performance during mealtime of the home care patients and feeding care provided by the caregivers were simultaneously observed and recorded. Among the six risk factors univariately correlated with the incidence of AP, feeding in a noisy environment, using a large spoon to feed the participants, more than 5 mL of food per mouthful, food intake duration lasting > 30 min, swallowing twice for each mouthful of food, and coughing at least once every day remained significant in the logistic regression model (all *p* < 0.05). Four risk factors for AP were correlated with feeding care; the adjusted risk ratio ranged from 6.17 to 14.96 (all *p* < 0.05). In addition to each individual’s food intake ability, improper feeding assistance was related to the risk factors for AP among home care patients with NGT-oral feeding. Thus, home caregivers should receive safe oral feeding education and training.

## 1. Introduction

Since 1995, home care services in Taiwan have played a crucial role in caring for individuals who cannot provide adequate self-care. Most patients with long-term care remain bedridden for a long time; their activities of daily living (ADL) are typically restricted to mobility around their home [1]. They often have various chronic diseases, clouded consciousness, severe disability, inadequate communication skills, inferior ADL ability, and high caregiver dependence [2]. The oral and physical functions of individuals requiring home care resemble those of healthy people. These functions gradually deteriorate with age; individuals requiring home care tend to develop dysphagia [3]. Dysphagia, common among home care patients, interferes with the oral intake of food [4] and often results in malnutrition, dehydration, weight loss, food reflux, aspiration pneumonia (AP), and even the risk of death.

In the past two decades, nasogastric tube (NGT) placement has become an indispensable medical procedure in acute and chronic medicine to maintain or supplement the nutrition and water supply of patients with cranial nerve injury, disease, and debilitating dysphagia [5,6]. NGT placement has become increasingly prevalent in Taiwan. According to statistics published by the Ministry of Health and Welfare [7], approximately 200,000 patients received NG intubation because of diseases and their sequelae, including aging and dementia. Patients aged ≥ 65 years accounted for 61.42% of those who received NGT placement [5]. Huang et al. [4] and Lin et al. [2] reported that 58% of home care patients and 65.66% of long-term care facility residents must rely on NGTs for food intake. Compared with the United States, Taiwan has a considerably higher usage of NGTs (5.8%) [8]. Thus, NG care has resulted in 47% of home care services in Taiwan.

The benefits of using nasogastric tube feeding (NGF) to resolve food intake difficulties remain controversial and inconsistent [8,9,10]. Research has proven that NGF is effective in improving nutritional status, survival rate, and lifespan [9,11]. By contrast, recent studies have reported that long-term use of NGF can neither reduce the risk of AP and pressure ulcers nor increase the nutritional status and survival rate of patients [8,9]. Furthermore, oral intake status is individually centered and highly correlated with their ability to swallow [12]. For example, only 20% of stroke survivors require full enteral feeding, although dysphagia is common [13]. Most NGF receivers are still capable of chewing and swallowing. The combination of feeding care with oral function enhancement and rehabilitation therapy can help stroke survivors transition from NGF to oral food intake [13].

Caregivers are frontline staff responsible for the daily life and diet of home care patients. Suzuki et al. [14] discussed the correlation between the survival rate and swallowing function of older patients with AP based on their 90-day mortality after swallowing function evaluation; significantly, more patients were judged to require food texture adjustment, meal assistance, and posture alterations in the deceased than in the survivors. Accordingly, patients with dysphagia who rely on oral food intake are vulnerable to hospitalization and even death from AP, especially if their caregivers lack adequate education and guidance regarding safe feeding practices. Therefore, this study explored the correlation between feeding care and AP risk among home care patients who rely on an in-dwelling NGT to complement oral food intake.

## 2. Methods

### 2.1. Study Design and Participants

This observational cross-sectional study recruited home care patients and their primary caregivers from a medical center with a home care center in southern Taiwan. This home care center provided home care services to 486 patients. There were 282 patients (58%) who had NGT placement. We used a telephone contact to confirm the willingness to participate in this study and excluded 154 patients due to living in long-term care facilities and patient and/or primary caregiver refusing to participate in this study. We visited the 128 patients and invited him/her and their caregiver to participate in this study together. We recruited 35 participants who had been hospitalized for AP in the past year.

All participants were diagnosed by a physician to require NG placement for dysphagia caused by central nervous system disease or injury, stroke, dementia, or Parkinson’s disease, etc. Participants with dysphagia relied on the NGT for food intake and received home care services from home care nurses. In this study, primary caregivers referred to those who took care of or managed the participants’ diet and food intake every day for more than 6 h for at least three consecutive months.

Home care services are covered by Taiwan’s National Health Insurance (NHI) [15,16]. According to the NHI payment regulations, patients eligible for home care services must fulfill the following criteria: (1) have limited self-care ability and are confined to a bed or a chair for ≥50% of their waking time; (2) clearly require a certain medical or nursing service; and (3) have a condition that is stable enough to receive home health care services, which are provided regularly by home care nurses from home care facilities during home care visits [16]. All participants in this study have received home care services for ≥3 years and had their medical history thoroughly recorded by a physician.

This study obtained the participants’ symptoms, signs, hospitalization details, and other medical history from their medical records. Clinical practice guidelines for nursing- and healthcare-associated pneumonia were used as the diagnostic scheme for AP [14,17]. After diagnostic confirmation with chest X-ray, patients were selected to be one of the participants of this study. For participants hospitalized for AP multiple times within a year, the latest AP hospitalization data were recorded.

### 2.2. Ethical Considerations

We obtained prior approval from the Chi-Mei Medical Center Institutional Review Board before conducting the study (No. 10405-009). All research procedures were performed in accordance with the ethical standards of the Chi-Mei Medical Center and National Research Council as well as the 1964 Declaration of Helsinki and its subsequent amendments or similar ethical standards. Before collecting data through an observation form to record their food intake performance and behavior, the principal investigator carefully explained the study aims and procedures to the participants with a clear consciousness, guardians of participants with unclear consciousness, and participant’s primary caregivers. The written informed consent was obtained from participants and their primary caregivers or from participant’s guardians and participant’s primary caregivers when both agreed to participate in this study.

### 2.3. Data Collection

#### 2.3.1. Questionnaire Collection

The observation form consisted of two sections of closed-ended items to collect data from the participants and their caregivers. The first section inquired about the participants’ demographic characteristics (i.e., age, sex, and education level), medical history, physical condition (i.e., chronic disease and state of consciousness), and dietary pattern (i.e., eating behavior and food types). The second section inquired about the caregivers’ demographic characteristics (i.e., age, sex, and education level), care experience, and experience of receiving instructions on safe feeding practices.

We conducted a retrospective case review to collect the participants’ medical records, demographic data, and physiological condition. Subsequently, caregivers were instructed to complete a structured self-report questionnaire, with the assistance of the same independent and well-trained researcher. Considering that some caregivers were foreign nationals, a translation agency was commissioned to translate the questionnaire into Indonesian and Vietnamese. The translation was then proofread by Indonesian, Chinese, and Vietnamese spouses in Taiwan before administration and they assisted the researcher to conduct the study on the same day.

The questionnaire content was evaluated for validity by an expert review team, which was comprised of five members with extensive knowledge and expertise related to oral function and dysphagia (i.e., family medicine physicians, speech therapists, dentists, long-term care specialists, and senior oral care nurses). The mean content validity index score was 0.96. To ensure the caregivers’ understanding of the questionnaire content, we pretested the questionnaire on 30 caregivers who were not included in this study.

#### 2.3.2. Observation of Oral Intake Performance and Assistance

The same home care nurse (one of the researchers: C.-C.C.) observed the process of all participants’ food intake and condition as well as their requirements for assistance to complete a meal. Specifically, the participants’ oral intake performance (e.g., independent eating ability, food type, ability of chewing food properly, coughing frequency during or after consuming food and/or liquid, swallowing frequency while swallowing per food mouthful, eating duration, and tableware), caregiver’s assistance with food intake (e.g., eating position), and other external factors (e.g., eating environment) were thoroughly evaluated.

### 2.4. Statistical Analysis

IBM SPSS Statistics 20.0 (IBM, Armonk, NY, USA) was used for all statistical analyses. Categorical demographic data were presented as numbers and percentages. The Fisher’s exact test was used to compare the food intake condition and relevant assistance behavior of the AP and non-AP groups.

Univariate and multiple logistic regression analyses were used to evaluate the correlation between unadjusted and adjusted food intake performance factors and the risk of pneumonia. Only when an independent variable was found to be significantly correlated with the risk of pneumonia incidence would it be included in the multiple logistic regression model. A Fisher’s exact test was used to compare the distribution of proportion of participants with AP between different groups of caregivers who received diet safety instructions. The significance level of all statistical tests was set at *p* < 0.05; the confidence interval (CI) was set at 95%.

## 3. Results

There were more men (65.71%) than women in this study. The participants tended to have clear consciousness (57.14%), were partially dependent on the assistance of others in their ADL (20.00%), were underweight (37.14%), and had a higher medical history of AP (62.90%) (Table 1). Four out of five of the caregivers were female (80.00%), aged over 45 years old (60%), had a level of education lower than senior high school (51.43%), not a relative of the patient (54.29%), and had care experience of less than 3 years (57.14%) (Table 2).

Table 3 indicates that the risk factors for the NGT–oral feeding group who had been hospitalized with AP included caregiver dependence for feeding, using a regular spoon for feeding, more than 5 mL of food per mouthful, swallowing twice for each mouthful of food, food intake duration lasting < 30 min, and feeding in a noisy environment. Participants with these characteristics had significantly higher chances of developing AP than those without these risk factors (all *p* < 0.05).

Subsequently, we used univariate and multiple logistic regression analyses to explore the risk of AP (Table 4). After adjustment for potential confounding factors (age, consciousness, disease, severity of disability), the participants who coughed at least once a day at mealtimes were significantly correlated with higher AP risks (AOR = 17.18, 95% CI = 2.51–11.71, *p* = 0.004). Caregivers’ experience of receiving instructions on safe feeding practices was significantly correlated with the participants’ AP incidence (Table 5). In terms of the experience of receiving instructions on safe feeding practice, the percentage of caregivers for patients with a medical history of AP (9.09%) was significantly lower than that of caregivers for patients without a medical history of AP (69.23%; *p* < 0.001). All caregivers of patients with a medical history of AP (100.00%) reported that they lacked adequate training in caring for patients with dysphagia (*p* = 0.001).

## 4. Discussion

This preliminary study explored the factors affecting the food intake condition of patients who had been hospitalized with AP in the past year and were receiving daily feeding care. All home care patients in this study received NGT placement due to dysphagia. These participants relied on both NGT and oral feeding for food intake and occupied 27.34% of the population with NGT placement, which concurs with the findings of Sugiyama et al. [18]; patients who have received NGT placement can resume oral intake and find pleasure in eating under the premise of high-quality care. Evidently, both internal (food intake function) and external factors (feeding care) could serve as risk factors that contribute to hospitalization for AP. We also discovered that caregivers of home care patients with a medical history of AP all reported that they lacked adequate training in safe swallowing; most of them had not received instructions on safe feeding practices. Therefore, safe feeding instructions may be provided to caregivers to help reduce the incidence of AP by a home care nurse while providing services.

Differences in the causes of NG placement in home care patients receiving different feeding methods may affect AP incidence. Approximately 60% of the patients had a medical history of stroke. In the NGT–oral feeding group, most members were male and relatively young. The numbers of participants diagnosed with dementia and Parkinson’s disease were 14.13% and 17.14%, respectively. Both dementia and Parkinson’s disease are common neurodegenerative diseases in older adults. We inferred that dysphagia in the NGT–oral feeding patients was attributable to disease sequelae, whereas dysphagia in the older adults was caused by both disease sequelae and aging. Members of the NGT–oral feeding patient group had clear consciousness and were partially dependent on the assistance of others for their ADLs. Furthermore, they contracted the disease at a young age. Thus, their quality of life was compromised due to the use of in-dwelling NGTs to change their body image, thereby leading to unsatisfactory eating conditions. Because NGT removal can help reverse these negative effects, some patients who had clear consciousness and adequate oral function were more willing to remove NGTs and transition to oral feeding. However, the incidence of AP in the NGT–oral feeding group (76.92%) was higher than that in the NGF group of previous study results (42.96%) [4], although the physical condition and oral function of the NGT–oral feeding group were superior to those of the NGF group. In this study, the results concur with those of [19,20], that is, orally fed patients had a higher AP incidence (54.3%) than tube-fed patients (13.2%). This implies that in addition to each individual’s own food intake and swallowing functions, their reception of quality care and assistance may affect the incidence of AP.

Although the NGT–oral feeding group had superior food intake and swallowing function than the NGF group, the occurrence of dysphagia should not be excluded. The swallowing and choking frequency are the two major causes of AP in home care patients with dysphagia. Furthermore, previous studies reported that the placement of NGT in individuals who rely on both NGT and oral feeding for food intake can interfere with swallowing as well as gastric and esophageal closure, thereby resulting in choking and increased AP risks [19,20]. In this study, the NGT-oral feeding group exhibited poor swallowing ability. Among the patients who consumed each mouthful of food by swallowing twice and choked at least once a day when swallowing food, approximately 70% were hospitalized with AP. This concurs with the findings of previous researchers who discussed AP-related factors and discovered that individuals who choke while eating tend to develop AP [19,20]. During the pharyngeal stage of swallowing, the unsmooth process of the bolus attempts to avoid the respiratory tract upon entry into the esophagus, thereby increasing the incidence of AP [21].

Inappropriate feeding methods affected AP risk in the NGT–oral feeding group. Home care patients with a medical history of AP lacked the ability to consume food on their own and fully relied on their caregivers. This aligns with the statement of Davison and Morrell [22], who reported that the most common comorbidity in individuals requiring feeding care was AP. Most eating environments in Taiwan have TV noises and images to induce a calming nature. Traditionally, the depth of the feeding spoon should be deeper; the amount of food per mouthful should exceed 5 mL. The aforementioned feeding care misaligns with existing feeding practices, including its management and suggested assistance. Consuming excessive amounts of food in each mouthful may easily cause choking, thus increasing AP incidence [14]. To exacerbate matters, a noisy eating environment can easily distract individuals from concentrating on chewing and swallowing, which also increases the risk of AP. Adopting an effective seated position can be utilized to complete a safe meal and reduce AP in patients with swallowing difficulties [23,24]. A seated position can raise the level of the patient’s consciousness, improve their mental alertness and reduce the incidence of aspiration [24]. Another research reported that adopting a 45° reclining position on swallowing is more useful for the patients with aspiration for small amounts of thin liquid and large amounts of residue in valleculae than adopting a 90° seated position [25]. We inferred that both a 45–60° reclining position or 90° seated position were beneficial for patients during mealtime and had no difference with the rate of AP.

Whether caregivers received instructions on safe feeding practices affected AP incidence in participants. Among the Taiwanese family caregivers in both groups, more than three-quarters were women [26]. Accordingly, AP incidence is affected by dysphagia severity, food intake function, and feeding care and assistance. Liu et al. [26] reported that education level is positively correlated with care knowledge, attitude, and skills. In the past year, patients taken care of by caregivers who lacked adequate training in safe swallowing practice had a higher AP incidence as compared with those taken care of by well-trained caregivers. Among the caregivers responsible for patients with a medical history of AP, only 9.09% had received safe feeding instructions, which is consistent with domestic and foreign literature results; most caregivers have not received formal education and training related to treating dysphagia in older adults [27,28]. All of the caregivers of patients with a medical history of AP expressed that they lacked adequate training in safe swallowing, which concurs with the results of Colodny [29], that caregivers’ lack of knowledge about treating dysphagia affected their attitude and confidence when handling patients with dysphagia. The results also highlighted the caregivers’ high demand for knowledge and skills related to dysphagia care. However, those with self-confidence tend to maintain a positive attitude and show higher self-efficacy in care. In this study, the caregivers of patients in the NGT–oral feeding group clearly required further training and guidance in dysphagia treatment to prevent the occurrence of comorbidities.

Efficient and safe feeding assistance is critical during mealtimes and should not be denied to patients with NGT-oral feeding. The caregivers in this study tended to apply inappropriate feeding practices. In the future, tips on safe feeding assistance must be taught to caregivers with a home care nurse providing service the first time. According to domestic and international literature, there are several strategies/tips for caregivers to reach a safe feeding assistance level, minimize the rise of choking and/or prevent AP risk. Before the meal, caregivers can select an environment with a quiet and homey atmosphere and provide texture-modified food and thickened fluids to meet the patients’ own unique needs. Caregivers should adjust and maintain a patient’s eating position of 45° reclined sitting posture, use one 5 mL teaspoon with the amount of food per mouthful to feed, and control eating duration within 30 min during mealtime to ensure a safe feeding plan and reduce the risk of aspiration [9,25,28,30,31,32].

This study has some limitations. First, because of the small sample size, we could not consider the potential effects of disease severity, physical disability, or mental disability. Further large-scale studies are warranted to validate our results and to increase the reliability of the conclusions. Care should be taken when extrapolating results to other home care facilities. Second, we did not clearly define the severity and/or stages of dysphagia in patients due to lack of further clinical evaluation (dental information, videoendoscopic and videofluorographic) of partial patients in this study, which made it difficult to understand the effect of dysphagia severity on the risk of AP incidence. Third, the effect of oral hygiene problems on AP risk could not be completely ruled out. Potential pathogenic microorganisms in the oral cavity may increase the bacterial load and oral colonization risks [4,33]. In this study, the possibility of oral bacteria increasing the risk of AP cannot be completely excluded. We did not record the type of nutrition prepared, such as the home prepared or other prepared products used and thickening products used. In Taiwan, most nutrition is home-prepared and one and/or two meals will use prepared products depending on the participants’ and/or their families’ financial ability. Using thickening products may increase diet safety. We cannot provide data on the usage of thickening products. A properly prepared and safe diet is important for home care patients. It is suggested to add the record of nutrition preparation, food textures and drink thickness in a future study, which should make a better interpretation of the research results.

Our findings revealed that compared with tube-fed patients who had no medical history of AP, the NGT–oral feeding group patients had a higher incidence of AP; they underwent improper food intake due to dysphagia. Improper feeding care by caregivers can further increase the incidence of AP.

## 5. Conclusions

Improper food intake and care can increase the incidence of AP. Providing caregivers with instructions on safe feeding practices may facilitate appropriate oral intake of food among home care patients, thereby reducing the risk of AP. Home care nurses are advised to provide primary home caregivers with proper education on safe feeding practice and oral feeding care, thus ensuring oral feeding safety among home care patients and preventing AP incidence.

## Figures and Tables

**Table 1:** Characteristics of participants.

Variable	*n*	(%)
Gender		
Male	23	65.7
Female	12	34.3
Age group		
<65 years old	9	25.7
≥65 years old	26	74.3
Marital status		
Married	34	97.1
Single	1	2.9
Education level		
Less than senior high school	28	80.0
Senior high school or higher	7	20.0
Chronic disease		
<3	15	42.9
≥3	20	57.1
Medical history ^a^		
Stroke	20	57.1
Dementia	13	14.1
Parkinson disease	6	17.1
Disability certificate		
Yes	26	74.3
No	9	25.7
Consciousness		
Clear	20	57.1
Unclear	15	42.9
Dependence in activities		
Completely	28	80.0
Partially	7	20.0
Weight status		
Underweight	13	37.1
Weight status	20	57.1
Overweight	2	5.8
Pneumonia		
No	13	37.1
Yes	22	62.9

^a^ Multiple choice.

**Table 2:** Characteristics of participants’ caregivers.

Variable	*n*	(%)
Gender		
Male	7	20.0
Female	28	80.0
Age group		
20–44 years old	14	40.0
45–64 years old	16	45.7
≥65 years old	5	14.3
Education level		
Less than senior high school	18	51.4
Senior high school or higher	17	48.6
Relationship with the cases		
Relative	16	45.7
Non-relative	19	54.3
Care experience		
<3 years	20	57.1
≥3 years	15	42.9

**Table 3:** Relationship between intake status and aspiration pneumonia of the participants of nasogastric feeding with combined oral feeding.

Variable	*N*	(%)	AP (*N* = 22)		Non-AP (*N* = 13)		*p*-Value
			*n*	(%)	*n*	(%)	
Eating ability							
Partially by feeding	4	(11.4)	0	(0.0)	4	(30.7)	0.014
Completely by feeding	31	(88.6)	22	(100.0)	9	(69.3)	
Coughing when drinhing liquid							
No	2	(5.7)	0	(0.0)	2	(15.4)	0.131
Yes	33	(94.3)	22	(100.0)	11	(84.6)	
Ability of chewing food properly							
Yes	18	(51.4)	9	(40.9)	9	(69.3)	0.105
No	17	(48.6)	13	(59.1)	4	(30.7)	
Swallowing frequency per food mouthful							
Once	18	(51.4)	7	(31.8)	11	(84.7)	0.005
Twice	17	(48.6)	15	(68.9)	2	(15.3)	
Choking frequency during or after consuming food and/or liquid							
Once a week	17	(48.6)	6	(27.3)	11	(84.6)	0.002
At least once a day	18	(51.4)	16	(72.7)	2	(15.4)	
Eating position							
Upright seated position (90°)	19	(54.3)	11	(50.0)	8	(61.5)	0.508
Reclining position (45–60°)	16	(45.7)	11	(50.0)	5	(38.5)	
Eating environment							
Noisy	26	(74.3)	19	(86.4)	7	(53.8)	0.019
Quiet and homey	9	(25.7)	3	(13.6)	6	(46.2)	
Food type							
Pureed diet	20	(57.1)	13	(59.1)	7	(53.9)	0.762
Finely chopped diet	15	(42.9)	9	(40.9)	6	(46.1)	
Tableware							
General spoon	20	(57.1)	17	(77.3)	3	(23.1)	0.015
Small teaspoon	15	(42.9)	5	(22.7)	10	(76.92)	
Food amount per mouthful							
>5 mL	13	(37.14)	12	(54.6)	1	(7.7)	0.010
≤5 mL	22	(62.86)	10	(45.4)	12	(92.3)	
Eating duration							
≤30 min	17	(5.71)	14	(63.6)	3	(23.1)	0.035
>30 min	18	(94.29)	8	(36.4)	10	(76.9)	

AP: aspiration pneumonia; Non-AP: non-aspiration pneumonia.

**Table 4:** Factors associated with aspiration pneumonia of the participants of nasogastric feeding with combined oral feeding.

Variable	COR ^a^	95%CI	*p*-Value	AOR ^b^	95%CI	*p*-Value
		(Lower, Upper)			(Lower, Upper)	
Tableware						
General spoon	1			1		
Small tea spoon	6.00	(1.33, 27.05)	0.020	11.23	(1.70, 74.22)	0.012
Eating environment						
Noisy	5.43	(1.06, 27.83)	0.043	6.17	(1.02, 37.26)	0.047
Quiet and homey	1			1		
Food amount per mouthful						
>5 mL	14.40	(1.59, 130.73)	0.018	14.96	(1.44, 155.21)	0.023
≤5 mL	1			1		
Swallowing frequency per food mouthful						
Once	1			1		
Twice	11.79	(2.04, 68.06)	0.006	15.21	(2.02, 114.59)	0.008
Choking frequency during or after consuming food and/or liquid						
At least once a day	14.67	(2.49, 86.53)	0.003	17.18	(2.51, 117.71)	0.004
Once a week	1			1		
Eating duration						
>30 min	1			1		
≤30 min	5.83	(1.23, 27.63)	0.026	13.01	(1.88, 89.93)	0.009

^a^ Crude odds ratios were derived from univariate logistic regression model. ^b^ Adjusted odds ratios were derived form a multiple logistic regression model mutually adjusted for age, gender, conscious, disease items, severity of disability.

**Table 5:** Relationship between feeding instruction experience of caregiver and aspiration pneumonia of the participants of nasogastric feeding with combined oral feeding.

Variable	*N*	AP (*N* = 22)		Non-AP (*N* = 13)		*p*-Value
		*n*	(%)	*n*	(%)	
The caregiver had received diet safety instructions						
Yes	11	2	(18.2)	9	(81.8)	<0.001
No	24	20	(83.3)	4	(16.7)	
The caregiver believe he/she had adequate training of dysphagia						
Yes	9	0	(0.00)	9	(100.0)	0.001
No	26	22	(84.6)	4	(15.4)	

AP: aspiration pneumonia; Non-AP: non-aspiration pneumonia.
